# Activation of Wnt/β‐catenin signalling is required for TGF‐β/Smad2/3 signalling during myofibroblast proliferation

**DOI:** 10.1111/jcmm.13085

**Published:** 2017-02-28

**Authors:** Liang Xu, Wen‐Hui Cui, Wen‐Cheng Zhou, De‐Lin Li, Liu‐Cheng Li, Ping Zhao, Xiao‐Ting Mo, Zhihui Zhang, Jian Gao

**Affiliations:** ^1^ The First Affiliated Hospital of Anhui Medical University Hefei China; ^2^ The Second Hospital of Dalian Medical University Dalian China; ^3^ School of Pharmacy Anhui Key Laboratory of Bioactivity of Natural Products Anhui Medical University Hefei China; ^4^ Taihe Hospital of Traditional Chinese Medicine Fuyang China; ^5^ The First Affiliated Hospital of Anhui Traditional Chinese Medicine University Hefei China

**Keywords:** Wnt/β‐catenin, pulmonary fibrosis, Smad2/3, myofibroblast proliferation

## Abstract

Fibrosis in animal models and human diseases is associated with aberrant activation of the Wnt/β‐catenin pathway. Despite extensive research efforts, effective therapies are still not available. Myofibroblasts are major effectors, responsible for extracellular matrix deposition. Inhibiting the proliferation of the myofibroblast is crucial for treatment of fibrosis. Proliferation of myofibroblasts can have many triggering effects that result in fibrosis. In recent years, the Wnt pathway has been studied as an underlying factor as a primary contributor to fibrotic diseases. These efforts notwithstanding, the specific mechanisms by which Wnt‐mediated promotes fibrosis reaction remain obscure. The central role of the transforming growth factor‐β (TGF‐β) and myofibroblast activity in the pathogenesis of fibrosis has become generally accepted. The details of interaction between these two processes are not obvious. The present investigation was conducted to evaluate the level of sustained expression of fibrosis iconic proteins (vimentin, α‐SMA and collagen I) and the TGF‐β signalling pathway that include smad2/3 and its phosphorylated form p‐smad2/3. Detailed analysis of the possible molecular mechanisms mediated by β‐catenin revealed epithelial–mesenchymal transition and additionally demonstrated transitions of fibroblasts to myofibroblast cell forms, along with increased activity of β‐catenin in regulation of the signalling network, which acts to counteract autocrine TGF‐β/smad2/3 signalling. A major outcome of this study is improved insight into the mechanisms by which epithelial and mesenchymal cells activated by TGFβ1‐smad2/3 signalling through Wnt/β‐catenin contribute to lung fibrosis.

## Introduction

Pulmonary fibrosis (PF) is a progressive interstitial lung disease characterized by fibroblast proliferation and excess deposition of extracellular matrix proteins, which lead to lung function distorted 1, 2, 3. Myofibroblasts are the major cells responsible for the production of collagens, and usually some of them are activated in the development of fibrosis. *In situ* transition of fibroblasts into α‐smooth muscle actin (α‐SMA)‐positive myofibroblasts, transdifferentiation of epithelial cells, tissue accumulation of bone marrow‐derived progenitor cells trafficking from the circulation are some of the putative mechanisms underlying the expansion of the pool of biosynthetically activated mesenchymal cells 4, 5. Therefore, a better understanding of the molecular mechanisms driving the genesis, progression and possible resolution of fibrosis is a prerequisite to the development of clinical therapies.

On the other hand, the possible mechanisms of myofibroblast elimination or dedifferentiation are still almost an uncharted territory 6, 7. Interstitial resident fibroblasts are considered to be the effective cells in the development of PF, and TGF‐β acts as a key factor which activation has been implicated in the fibrosis of both PF and airway remodelling in this process 8, 9. To be universally known, TGF‐β action is highly context dependent and influenced by cell type, culture conditions, and interaction with other signalling pathways, developmental or disease stage *in vivo* 7. In recent years, more and more studies show that TGF/smad signalling pathway plays an important role in the disease of fibrosis 10, 11.On the contrary, if TGF‐β targeted for interments fibrosis, many side effects will appear simultaneously. For this reason, TGF‐β unfortunately declared not an ideal target in the fibrotic diseases 9, that is an urgent need to find other targets in the fibrotic diseases 12.

Smads family proteins play a key role in the process of the transfer of TGF‐beta signalling from cell surface receptors to the nucleus, and signal transduction mediated by different Smad and different TGF family members 13. There are eight distinct Smad proteins, constituting three functional classes: the receptor‐regulated Smad, the co‐mediator Smad and the inhibitory Smad. The activated Smad complexes are translocated into the nucleus and, in conjunction with other nuclear cofactors, regulate the transcription of target genes. Fibrosis‐derived mutations have been observed in both TGF‐β family receptors and the Smad proteins. Although it appears that epithelial cell activation of TGF‐β by the smads is central in PF, mesenchymal activation of TGF‐β by the smad2 and smad3 could predominate in myofibroblast proliferation is still remains controversial 14, 15.

Canonical Wnt/β‐catenin signalling is one of the major oncogenic pathways that provide essential developmental signals during embryogenesis. Meanwhile, the importance of dysregulated Wnt/β‐catenin signalling in a variety of human diseases has been long appreciated 16. In addition, several cases have demonstrated a functional interaction between the canonical Wnt/β‐catenin and TGF‐β signalling pathways 17, 18. Whereas there is evidence for crosstalk between these two pathways in the context of development and tumorigenesis, effects of these interactions in non‐transformed adult cells are not well defined 19, 20. Precise targets and mechanisms about the divergent functional outcome of these interactions in many cases remain unknown.

In this study, we used alveolar epithelial cell (AEC) and fibroblasts to investigate TGF‐β/β‐catenin pathway convergence, as well as its consequences on transcription of α‐SMA and collagen I, target genes associated with myofibroblast proliferation. This article describes the mechanisms through which epithelial cells and mesenchymal cells activated by TGFβ1‐smad2/3 signalling through Wnt/β‐catenin and highlights its role in lung fibrosis. Understanding the mechanism on the basis of myofibroblast proliferation may provide useful strategies for preventing or reducing fibrosis.

## Materials and methods

### Cell line and culture

Human pulmonary epithelial cell line A549 and human embryonic lung fibroblast HELF were obtained from ATCC (Manassas, VA, USA) and cultured in 1640 medium (Gibco, NY, USA) in the presence of 10% foetal bovine serum (FBS; Gibco) at 37°C in a humidified atmosphere with 5% CO_2_. When the cells reached 70–80% confluence and then cultured in 1640 medium without FBS for 6 hrs before stimulated with recombinant human TGF‐β1 (100‐21C; PeproTech, Princeton, USA) for 24 hrs. Then, treated with ICG‐001 10 μM (S266202; Selleck, Houston, USA) for 6 hrs was added to the medium at the indicated concentrations and time‐points. Nuclear and cytoplasmic protein extracts from A549 and HELF cells for Western blot analysis.

### β‐catenin siRNA transfection

For β‐catenin knockdown, A549 and HELF cells were transfected with small‐interfering RNA (siRNA) targeting the human β‐catenin gene or control non‐targeting siRNA using lipofectamine 2000 (Invitrogen, Carlsbad, CA, USA). The β‐catenin siRNA and negative control siRNA were designed and synthesized by GenePharma (Shanghai, China) according to human‐specific sequences. The primer sequences of β‐catenin siRNA were as follows: β‐catenin forward, 5′‐GCAGUUGUAAACUUGAUUATT‐3′, β‐catenin reverse, 5′‐UAAUCAAGUUUACAACUGCTT‐3′, which were transfected into A549 and HELF with lipofectamine 2000 according to the manufacturer's instructions. To identify whether the gene is silenced completely, reverse‐transcription polymerase chain reaction (RT‐PCR) was used. Total mRNA was extracted from cells using TRIzol reagent (Invitrogen) and cDNA was synthesized from mRNA using Prime Script RT‐PCR synthesis kit (RR036A; TaKaRa, Otsu, Shiga, Japan) following the manufacturer's instructions. RT‐PCR was performed under standard protocol. The following primers were synthesized by Sangon Biotech Co., Ltd (Shanghai, China): β‐catenin, the forward primer was 5′‐GGGTCCTCTGTGAACTTGCT‐3′ and the reverse was 5′‐AATCTTGTGGCTTGTCCTCA‐3′, and the amplicon size was 162 bp. β‐actin, the forward primer was 5′‐TCAGGTCATCACTATCGGCAAT‐3′ and the reverse was 5′‐AAAGAAAGGGTGTAAAACGCA‐3′, and the amplicon size was 432 bp. Results were presented as relative mRNA levels normalized against those of β‐actin. After 6 hrs, medium was replaced with fresh medium supplemented with or without TGF‐β1 for an additional 24 hrs. Proteins were harvested for detection by Western blot analysis.

### Animals and treatments

Forty Sprague Dawley rats weighing 180–220 g were purchased from the Experimental Animal Center of Anhui Medical University, Hefei, Anhui, China, and assigned to two groups randomly. Lung injury in the model group was induced by the endotracheal injection of 5 mg/kg bleomycin (BLM; Laiboten Pharmaceutical Co., Ltd, Harbin, China), while the control group received the same volume of saline instead. The day of intratracheal injection with BLM or saline was designated day 0. At 14 and 28 days after BLM injection, the rats were anaesthetized with 10% chloral hydrate (2.5 ml/kg) intraperitoneally, and then the animals were killed and the lungs were removed. All rats experimental procedures were approved by Anhui Medical University Animal Care Committee, and followed the protocol outlined in The Guide for the Care and Use of Laboratory Animals published by the USA National Institute of Health (NIH Pub. No. 85–23, Revised 1996).

### Histopathologic assessment and immunohistochemical staining

H&E and immunohistochemical (IHC) staining were performed as described previously 21.

### Western blot analysis

Lung homogenates and cell lysates were collected in RIPA Lysis Buffer (P0013C; Beyotime, Shanghai, China) containing 1 mM proteinase inhibitor phenylmethylsulfonyl fluoride (PMSF; Amresco 0754, Biosharp, Hefei, China). Nuclear and Cytoplasmic Protein Extraction Kits were used to collect protein in the nuclear (P0028; Beyotime). The extracts were mixed with sample buffer in a ratio of 4:1 and denatured by boiling. Then, the equivalent amount of protein was separated using 12% SDS‐PAGE and transferred to PVDF membranes (IPVH00010; Millipore, Billerica, MA, USA). After blocked with 5% non‐fat milk (Guangming, China) for 2 hrs, the membranes were incubated overnight at 4°C with the primary antibodies anti‐E‐cadherin (ab76055, 1:1000; Abcam, Cambridge, UK), anti‐α‐SMA (ab5694, 1:300; Abcam, Cambridge, UK), anti‐vimentin (ab92547, 1:5000; Abcam, Cambridge, UK), anti‐Col‐I (ab34710, 1:1000; abcam, Cambridge, UK), anti‐Smad2/3 (sc‐8332, 1:200; Santa Cruz), anti‐p‐Smad2/3 (sc‐11769, 1:500; Santa Cruz, Dallas, USA), anti‐β‐catenin (ab32572, 1:5000; Abcam, Cambridge, UK), anti‐H3 (ab1791, 1:5000; Abcam, Cambridge, UK), anti‐GAPDH (AP0063, 1:5000; Bioworld, Nanjing, China) and anti‐β‐actin (ab52614, 1:5000; Abcam, Cambridge, UK) antibodies, which were all from USA. On the next day, the blots were incubated with anti‐rabbit or antimouse IgG horseradish peroxidase‐conjugated secondary antibodies (ZSGB‐BIO, Beijing, China) for 1 hr at room temperature. Finally, the signal was detected with enhanced chemiluminescence reagent (ECL; Thermo Scientific, Rockford, IL, USA). Intensity was quantified using Scion Image software (Media Cybernetics, Inc., Rockville, MD, USA). All experiments were performed independently at least three times. β‐actin or GAPDH was used as an equal protein loading control.

### Statistical analysis

Data are expressed as the mean ± standard deviation (S.D.). Differences in measured variables between experimental and control groups were assessed using Student's *t‐*test. Groups containing multiple comparisons were analysed using anova, and values of *P* < 0.05 were considered to be statistically significant.

## Results

### Aberrant Wnt/β‐catenin activation in PF

**Figure 1:**
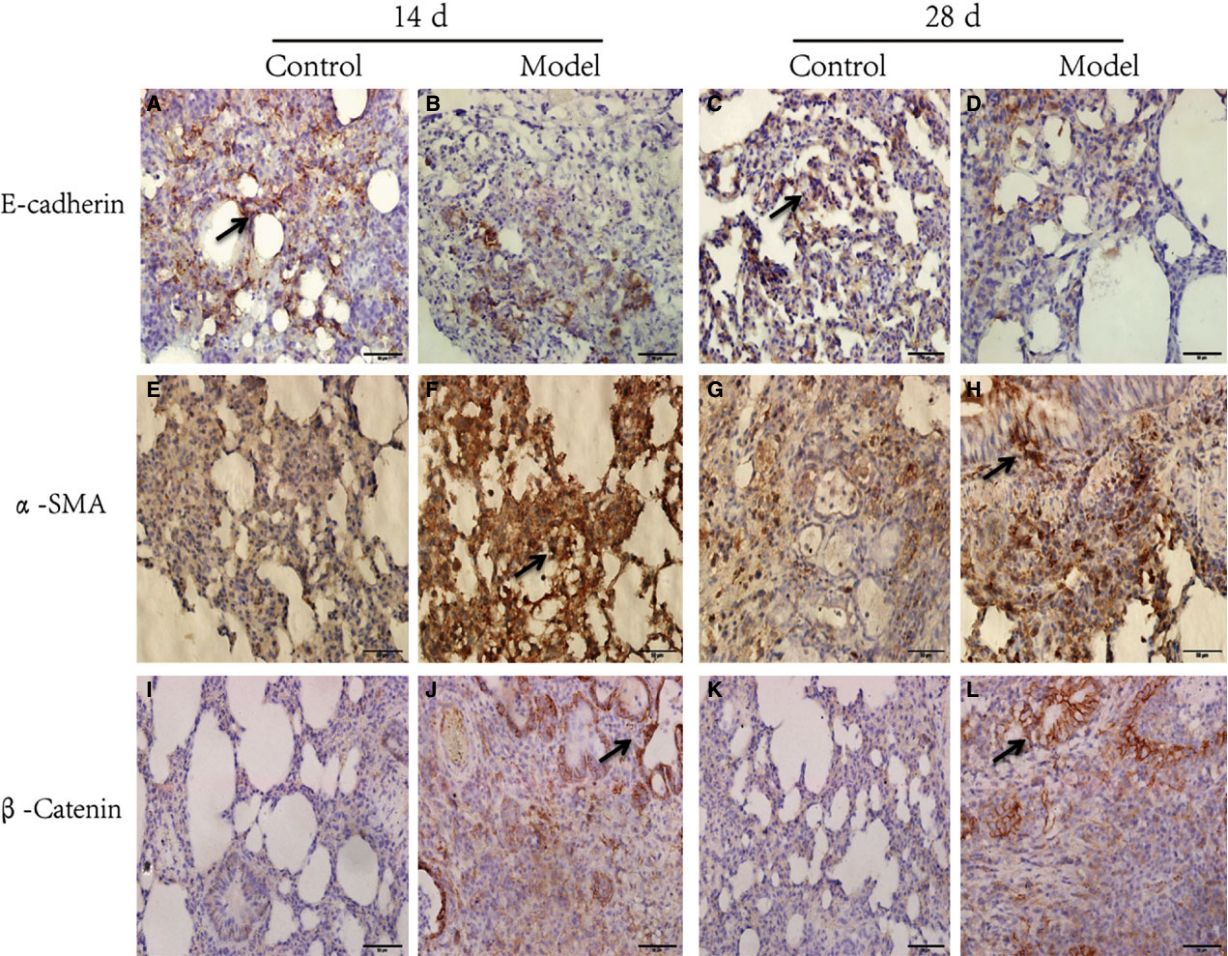
Immunohistochemical analysis of E‐cadherin, α‐SMA and β‐catenin in pulmonary fibrosis rat model. Representative images of lung biopsy specimens obtained from rats with pulmonary fibrosis and healthy control subjects. Top two lines, arrows indicate E‐cadherin‐positive alveolar epithelial cell just in normal lung tissues and α‐SMA‐positive fibroblast‐like cells in the pulmonary alveolus in bleomycin‐injured rats. Last line, arrows indicate nuclear β‐catenin‐positive fibroblast‐like cells in the pulmonary alveolus. *n* = 5 bleomycin‐ and saline‐treated mice. Original magnification ×400.

To evaluate myofibroblast proliferation *in vivo*, 40 rats received intratracheal injection of saline ± bleomycin (5 mg/kg). After 14 and 28 days, immunostaining of lung paraffin section with E‐cadherin (Fig. 1A–D) and α‐SMA (Fig. 1E–H) was performed. Expression α‐SMA was observed on days 14 and 28 after administration of bleomycin, but not in saline‐treated mice. Meanwhile, expression of E‐cadherin was markedly reduced in the pulmonary alveolus in bleomycin‐injured rats. Furthermore, to gain insight into the potential significance of the Wnt/β‐catenin axis in PF, we examined the expression of β‐catenin accompanied by a fibrotic reaction. As shown clearly, immunohistochemical analysis demonstrated that bleomycin induced rapid nuclear accumulation of β‐catenin in these fibroblasts (Fig. 1I–K).

### TGF‐β1 activates fibroblast‐to‐myofibroblast transition and epithelial–mesenchymal transition to induce α‐SMA expression

**Figure 2:**
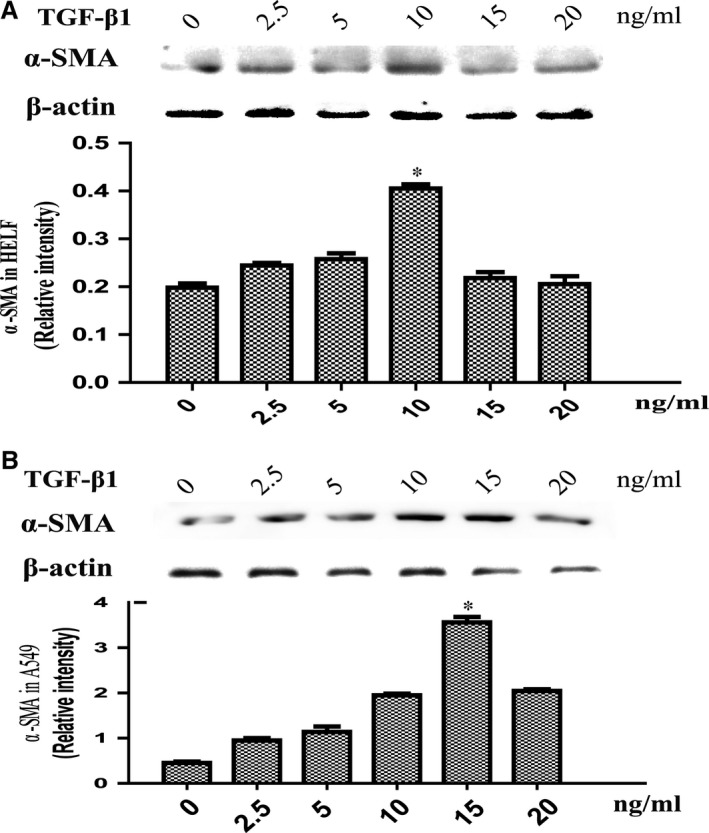
Western blot was used to examine the protein expression of α‐SMA in HELF (**A**) and A549 (**B**) cells exposed to the indicated doses of TGF‐β1 for 24 hrs.

Because of TGF‐β1, a pivotal trigger for profibrotic responses has been implicated in myofibroblast differentiation. To evaluate whether TGF‐β1‐mediated myofibroblast transdifferentiation, we first investigated a dose‐dependent increase in α‐SMA expression in fibroblasts and AEC stimulated with recombinant TGF‐β1. HELF and A549 cells were treated with TGF‐β1 for 24 hrs. As shown in Figure 2A and B, TGF‐β1 increased α‐SMA expression by nearly two times compared with the control group at 10 ng/ml for HELF, and almost three times increased at 15 ng/ml for A549.

### *TGF‐*β1 induced stimulation of profibrotic responses in normal fibroblasts

**Figure 3:**
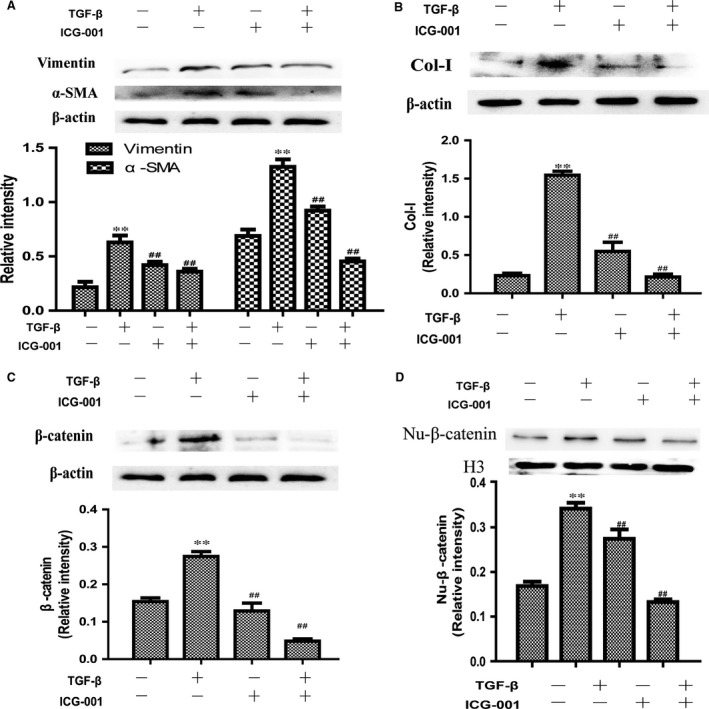
Effect of ICG‐001 exposure on the expression of vimentin, α‐SMA and collagen I in HELF cells. (**A** and **B**) Marker protein of fibroblast‐to‐myofibroblast transition vimentin, α‐SMA and collagen I overexpression in HELF cells caused by TGF‐β1 for 24 hrs. (**C**) Total protein of β‐catenin and (**D**) positive‐β‐catenin protein in the nuclear was tested by Western blot. Data are shown as means ± S.D., (*n* = 3). **P < 0.01, compared with control group. ##*P* < 0.01, compared with model group.

Fibroblasts are now recognized as the key effector cells in the development of fibrosis disease. To determine whether TGF‐β1 could activate fibroblasts by direct interaction with Wnt/β‐catenin signalling, we selected HELF fibroblasts and pre‐treatment with ICG‐001(10 μM) for 4 hrs before treated them with TGF‐β1 (10 ng/ml) for 24 hrs. As shown in Figure 3A and B, TGF‐β1 caused vimentin, α‐SMA and collagen I overexpression. In ICG‐001 intervention group, this increase is significantly suppressed. At the same time, total protein of β‐catenin (Fig. 3C) and positive‐β‐catenin protein in the nuclear (Fig. 3D) were significantly decreased in ICG‐001 intervention group in contrast with TGF‐β1 simple stimulation group in HELF cells.

### *TGF‐*β1 induced stimulation of epithelial–mesenchymal transition

**Figure 4:**
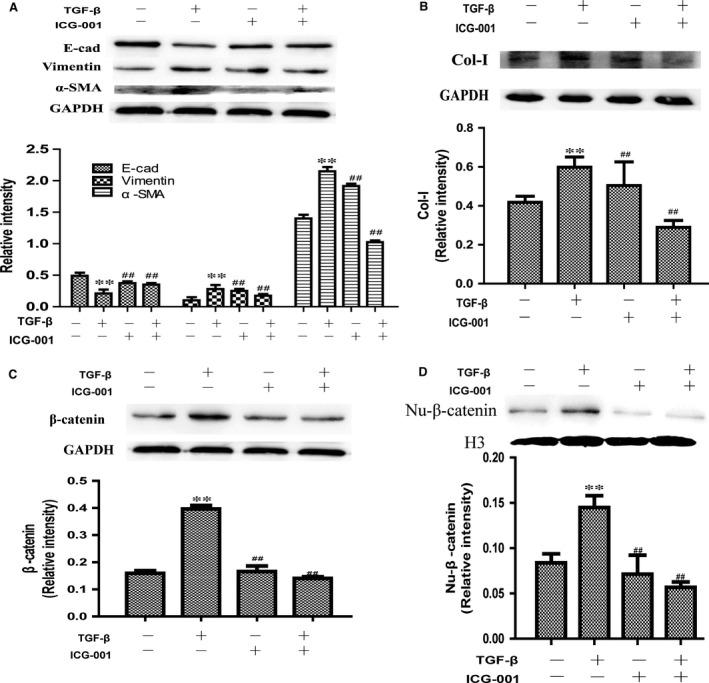
Effect of ICG‐001 exposure on the expression of E‐cad, vimentin, α‐SMA and collagen I in A549 cells. (**A** and **B**), Marker protein of epithelial–mesenchymal transition E‐cadherin vimentin, α‐SMA and collagen I overexpression in A549 cells caused by TGF‐β1 for 24 hrs. (**C**) Total protein of β‐catenin and (**D**) positive‐β‐catenin protein in the nuclear was tested by Western blot. Data are shown as means ± S.D., (*n* = 3). **P < 0.01, compared with control group. ##*P* < 0.01, compared with model group.

Several studies have demonstrated that alveolar type‐II epithelial cells in response to TGF‐β can undergo the EMT and differentiate into myofibroblasts 5, 15. Loss of E‐cadherin, the prototypic epithelial adhesion molecule in adherens junctions, and gain of α‐SMA have been recognized as main hallmarks of the EMT. Therefore, to evaluate whether TGF‐β1‐mediated EMT involves crosstalk with Wnt/β‐catenin signalling, we first investigated synergistic interactions between TGF‐β1 and Wnt/β‐catenin pathways in induction of EMT in A549 cells. With ICG‐001 administrated, suppressed α‐SMA, vimentin and collagen I overexpression and inhibited the loss of E‐cadherin caused by TGF‐β1 exposure in this process (Fig. 4A and B). Furthermore, protein of nuclear β‐catenin‐positive cells in the pulmonary alveolus were quantified as described in Patients and Methods. Total protein of β‐catenin (Fig. 4C) and positive‐β‐catenin protein in the nuclear (Fig. 4D) were significantly decreased in ICG‐001 intervention group in contrast with TGF‐β1 simple stimulation group in A549 cells.

### Role of canonical Wnt signalling in mediating TGF‐β1‐induced profibrotic responses

**Figure 5:**
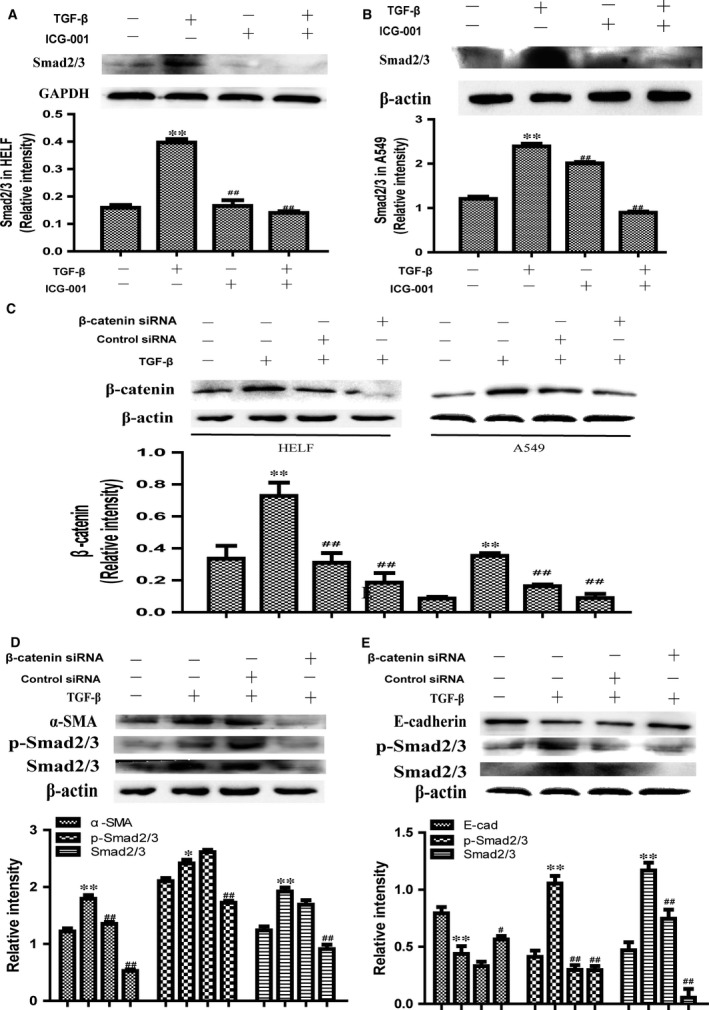
TGF‐β1‐induced fibrotic reaction is Smad dependent. ICG‐001 inhibits TGF‐β1‐induced Smad2/3 expression fibroblast induction and EMT, and representative Western blot and quantitative analysis of total Smad2/3 in lysate from HELF (**A**) and A549 (**B**) cells treated with ICG‐001. GAPDH or β‐actin is used as a loading control (*n* = 3; **P < 0.01 compared with control group; ##*P* < 0.01 compared with TGF‐β1 in the absence of β‐catenin siRNA). **C)** Representative Western blot and quantitative analysis of total β‐catenin in lysate from A549 and HELF cells transfected with β‐catenin or control siRNA followed by TGF‐β1treatment for 24 hrs. The expression of β‐catenin was detected by Western blots. β‐actin is used as a loading control (*n* = 3; **P < 0.01 compared with control group; ##*P* < 0.01 compared with TGF‐β1 in the absence of β‐catenin siRNA). Representative Western blot and quantitative analysis of α‐SMA, E‐cadherin, p‐Smad2/3 and Smad2/3 in lysate from A549 (**D**) and HELF (**E**) cells transfected with β‐catenin or control siRNA followed by TGF‐β1treatment for 24 hrs. β‐actin is used as a loading control (*n* = 3; **P < 0.01 compared with control group; ##*P* < 0.01 compared with TGF‐β1 in the absence of β‐catenin siRNA).

Having established that TGF‐β1 can induce the expression of fibroblast‐to‐myofibroblast transition and epithelial–mesenchymal transition markers such as α‐SMA, vimentin, collagen I and E‐cadherin, we next explored possible mechanisms underlying the TGF‐β1‐induced myofibroblast proliferation. The ability of TGF‐β1/Smad2/3 signalling to stimulate myofibroblast proliferation and the extracellular matrix production by cultured AECs and fibroblasts *in vitro* is well documented. To evaluate the effect of β‐catenin on activating TGF‐β1/Smad2/3 signalling, A549 and HELF cells were exposed to a constant dose of ICG‐001 (10 μM) for the indicated times. The results show that the expression of smad2/3 proteins inhibited by ICG‐001 significantly (Fig. 5A and B).

To further investigate the mechanisms whereby these two signalling pathways may interact in the context of fibroblast‐to‐myofibroblast transition and epithelial–mesenchymal transition, we used siRNA to knock down β‐catenin expression in TGF‐β1‐treated A549 and HELF cells. Cell lysates were examined by Western blot analysis for the activation of Smad2/3 using phosphospecific antibodies. The ‘p‐Smad2/3′ means phospho‐Smad2/3 (the phosphorylated Smad2/3), and the ‘Smad2/3′ indicates the total Smad2/3 protein (including the p‐Smad2/3 and the Smad2/3 not phosphorylated in the cells). The results show that TGF‐β1 directly activates Smad2/3 phosphorylation and β‐catenin siRNA effectively inhibits the activation of Smad2/3 (Fig. 5D and E).

## Discussion

Pulmonary fibrosis is characterized by excessive proliferation of fibroblasts and concomitant collagen accumulation within the alveolar and interstitial compartments of the lung 22. Myofibroblasts are the major cells responsible for the production of collagen, and usually some of them are activated during fibrotic reaction. However, the corresponding underlying mechanisms about alveolar type‐II epithelial cells and fibroblasts transited to myofibroblasts remain largely unaddressed 8. For these reasons, the conclusions from data obtained with the PF model should be, when considered from the perspective of the human diseases, understood as possible mechanisms that should be checked, but might not apply to all myofibroblast‐ sources‐like cells in each particular tissue environment. Here, we found that both fibroblasts and epithelial cells in the lungs could directly transform to myofibroblasts. Nevertheless, it has not been elucidated the sources and mechanisms of the myofibroblast proliferation. Thus, to really ease the process of lung fibrosis, blocking the EMT pathway alone is not enough. In our work, we not only present both of the two processes in our work, but also unearth the linked signalling. We showed that both epithelial cells and mesenchymal cells were activated by TGFβ1‐smad2/3 signalling through Wnt/β‐catenin. Understanding these events may provide novel perspectives for preventing or reducing fibrosis, while Wnt/β‐catenin pathway may provide promising targets for antifibrotic therapeutic approaches.

**Figure 6:**
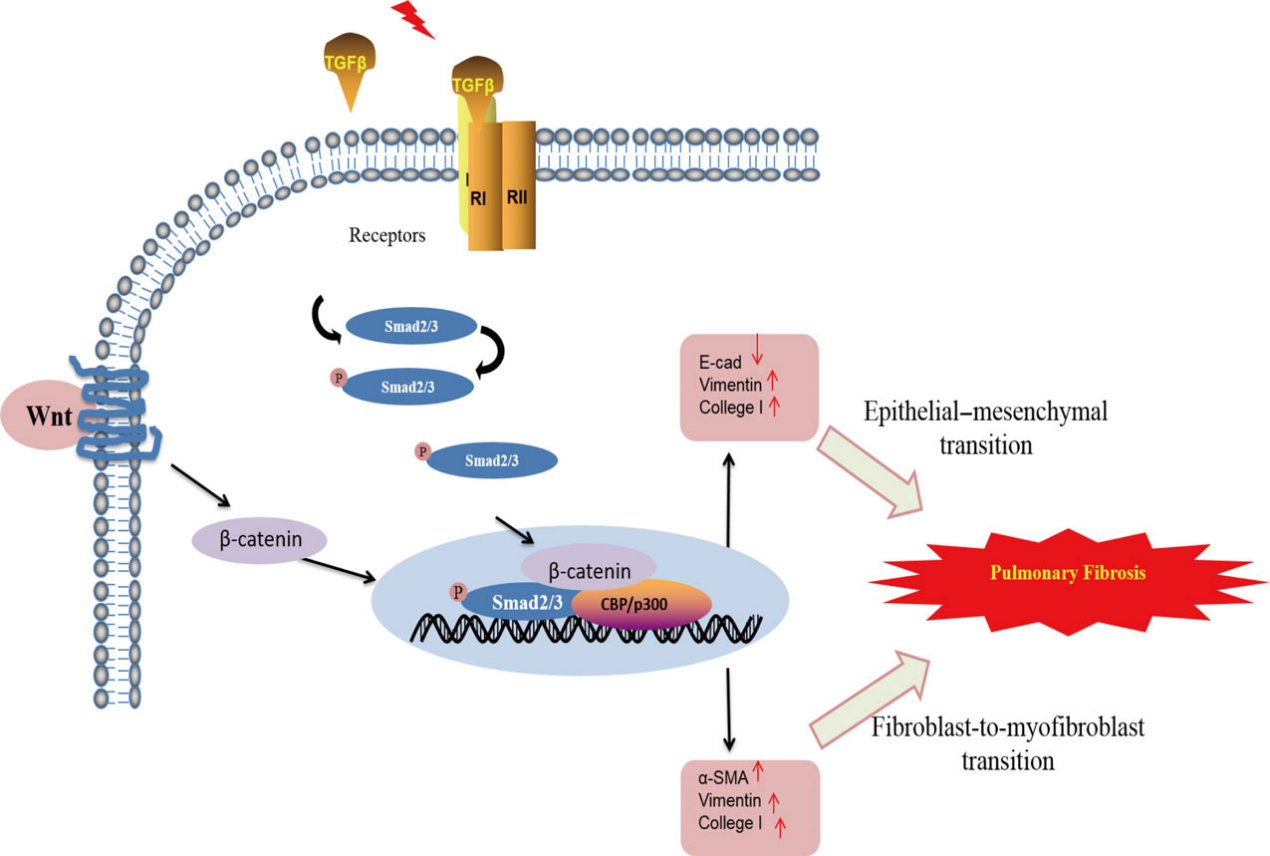
β‐catenin promotes the fibroblast‐to‐myofibroblast transition and EMT through the activation of the TGF‐β1/Smad2/3 signalling pathway. In general, TGF‐β/Smad2/3 signalling is initiated with TGF‐β‐induced phosphorylation of the cytoplasmic signalling molecules Smad2/3. The phospho‐Smad2/3 (p‐Smad2/3) combines with β‐catenin and translocates to the nucleus, where it will modulate the transcription of several target genes by binding with cyclic AMP‐responsive element‐binding protein (CREB)‐binding protein (CBP) to DNA sequences. Our findings suggest that β‐catenin promote both basal and TGF‐β1‐induced phosphorylation of Smad2/3.

In the present study, we investigated the direct interaction of TGF‐β1 with fibroblasts or epithelial cells, and the potential cellular effects caused by TGF‐β1 in fibroblasts or epithelial cells. Although the present data show that TGF‐β1 directly induced the phosphorylation of Smads, the mechanism underlying TGF‐β1‐activated phosphorylation of Smads is still unresolved. However, it has not been elucidated whether β‐catenin involves the recognized TGF‐β1/Smad2/3 signalling that contributes to myofibroblast proliferation in PF 23. As summarized in Figure 6, the present data suggest that β‐catenin promotes fibroblast activation and the EMT through the activation of the TGF‐β1/Smad2/3 signalling pathway. ICG‐001, a novel small molecule inhibitor of TCF/β‐catenin‐dependent transcription, inhibits β‐catenin/cyclic AMP‐responsive element‐binding protein (CREB)‐binding protein (CBP) but not β‐catenin/p300 interactions specifically 24. In this study, ICG‐001 prevented induction of α‐SMA and collagen I in lung epithelial cells in response to TGF‐β1, suggesting that β‐catenin/CBP interaction is absolutely required for regulation of at least a subset of mesenchymal genes generated by TGF‐β1 and may be critically important in the pathogenesis of EMT and fibrosis. Furthermore, we have demonstrated that a combination of chemical inhibition of Wnt/β‐catenin signalling and a perturbation of TGF‐β1/smad2/3‐signalling network with β‐catenin siRNA together induce a pronounced myofibroblast dedifferentiation.

Our results indicate that TGF‐β1 is a multifunctional cytokine that plays important roles in the fibroblast‐to‐myofibroblast transition and EMT. The canonical TGF‐β signalling pathway involves TGF‐β receptor‐mediated phosphorylation of the Smad proteins including Smad2 and Smad3, which dimerize together, and after phosphorylation translocate to the nucleus to induce expression of target genes. As is stated above, the new target for antifibrotic urgent need to find except for targeting TGF‐β1. Although dysregulation of Wnt signalling is implicated in tumorigenesis, the potential relevance of Wnt signalling in fibrogenesis has only recently begun to be appreciated, and the potential mechanisms remain to be elucidated. Accumulated data have shown that canonical Wnt/β‐catenin pathway is involved in the induction of myofibroblast proliferation 25, 26, 27. Wnt pathway activation promoted myofibroblast differentiation *via* Smad‐dependent TGF‐β signalling while suppressing AECs or fibroblast differentiation and inducing their differentiation into myofibroblasts. The net effect of these combined stimulatory and inhibitory activities is to promote fibrogenesis 20. That is to say, targeting Wnt pathway seems to be a good choice in fibrotic process 28, 29, 30. Resulting status of the signalling network we use the term perturbation here may not be described as simple activation or repression and as we have shown above (Fig. 5), expression of smad2/3 protein is suppressed by treated with ICG‐001 during TGF‐β1/smad2/3 signalling activation. These observations provide further support for the pivotal role of aberrant Wnt/β‐catenin pathway in various forms of fibrosis and indicate the feasibility of targeting Wnt signalling to prevent or reverse the fibrotic process.

In summary, the demonstration that impaired Wnt antagonism is associated with Wnt/β‐catenin pathway hyperactivation in lung tissue obtained from a subset of rats with PF. Canonical Wnt/β‐catenin signalling involved in fibroblasts and AECs stimulated their proliferation, migration and myofibroblast proliferation. Induction of interaction between β‐catenin by TGF‐β and inhibition of this interaction and of myofibroblast proliferation by ICG‐001 and β‐catenin siRNA suggest that β‐catenin is required for regulation of fibrotic reaction by Smad2/3. Therefore, the Wnt/β‐catenin pathway is a promising target for antifibrotic therapeutic approaches. The exact molecular mechanism responsible for the repression of expression of contractility‐related genes after Wnt/β‐catenin signalling perturbation in myofibroblasts is unknown and its elucidation represents an attractive challenge for future researches.

## Author contributions statement

Liang Xu, L.C.Li participated in research design; L.C.Li, D.L.Li and Zhihui Zhang conducted experiments; X.T.Mo, W.C.Zhou, P.Zhao contributed new reagents or analytic tools; L.C.Li, W.H.Cui, X.T.Mo performed data analysis; Liang Xu, J.Gao wrote or contributed to writing of the manuscript.

## Conflict of interest

All authors disclose that they do not have any commercial association that might pose a conflict of interest in connection with the manuscript.

## References

[1] Wynn TA , Ramalingam TR . Mechanisms of fibrosis: therapeutic translation for fibrotic disease. Nat Med. 2012; 18: 1028–40. 22772564 10.1038/nm.2807 PMC3405917

[2] Rydell‐Tormanen K , Andreasson K , Hesselstrand R , *et al* Extracellular matrix alterations and acute inflammation; developing in parallel during early induction of pulmonary fibrosis. Lab Invest. 2012; 92: 917–25. 22469699 10.1038/labinvest.2012.57

[3] Dempsey OJ , Miller D . Idiopathic pulmonary fibrosis. BMJ. 2013; 347: f6579. 24201334 10.1136/bmj.f6579

[4] Tomasek JJ , Gabbiani G , Hinz B , *et al* Myofibroblasts and mechano‐regulation of connective tissue remodelling. Nat Rev Mol Cell Biol. 2002; 3: 349–63. 11988769 10.1038/nrm809

[5] Chapman HA . Epithelial‐mesenchymal interactions in pulmonary fibrosis. Annu Rev Physiol. 2011; 73: 413–35. 21054168 10.1146/annurev-physiol-012110-142225

[6] Artaud‐Macari E , Goven D , Brayer S , *et al* Nuclear factor erythroid 2‐related factor 2 nuclear translocation induces myofibroblastic dedifferentiation in idiopathic pulmonary fibrosis. Antioxid Redox Signal. 2013; 18: 66–79. 22703534 10.1089/ars.2011.4240

[7] Gressner AM , Weiskirchen R . Modern pathogenetic concepts of liver fibrosis suggest stellate cells and TGF‐beta as major players and therapeutic targets. J Cell Mol Med. 2006; 10: 76–99. 16563223 10.1111/j.1582-4934.2006.tb00292.x PMC3933103

[8] Wolters PJ , Collard HR , Jones KD . Pathogenesis of idiopathic pulmonary fibrosis. Ann Rev Pathol. 2014; 9: 157–79. 24050627 10.1146/annurev-pathol-012513-104706 PMC4116429

[9] Su BH , Tseng YL , Shieh GS , *et al* Over‐expression of prothymosin‐alpha antagonizes TGFbeta signalling to promote the development of emphysema. J Pathol. 2016; 238: 412–22. 26496995 10.1002/path.4664

[10] Akhurst RJ , Hata A . Targeting the TGFbeta signalling pathway in disease. Nat Rev Drug Discovery. 2012; 11: 790–811. 23000686 10.1038/nrd3810 PMC3520610

[11] Oh CJ , Kim JY , Min AK , *et al* Sulforaphane attenuates hepatic fibrosis *via* NF‐E2‐related factor 2‐mediated inhibition of transforming growth factor‐beta/Smad signaling. Free Radic Biol Med. 2012; 52: 671–82. 22155056 10.1016/j.freeradbiomed.2011.11.012

[12] Haines DD , Lekli I , Teissier P , *et al* Role of haeme oxygenase‐1 in resolution of oxidative stress‐related pathologies: focus on cardiovascular, lung, neurological and kidney disorders. Acta Physiol. 2012; 204: 487–501. 10.1111/j.1748-1716.2011.02387.x 22118298

[13] Shi Y , Massague J . Mechanisms of TGF‐beta signaling from cell membrane to the nucleus. Cell. 2003; 113: 685–700. 12809600 10.1016/s0092-8674(03)00432-x

[14] Wang P , Wang Y , Nie X , *et al* Multiwall carbon nanotubes directly promote fibroblast‐myofibroblast and epithelial‐mesenchymal transitions through the activation of the TGF‐beta/Smad signaling pathway. Small. 2015; 11: 446–55. 25255886 10.1002/smll.201303588

[15] Wang P , Nie X , Wang Y , *et al* Multiwall carbon nanotubes mediate macrophage activation and promote pulmonary fibrosis through TGF‐beta/Smad signaling pathway. Small. 2013; 9: 3799–811. 23650105 10.1002/smll.201300607

[16] Lv ZD , Yang ZC , Liu XP , *et al* Silencing of Prrx1b suppresses cellular proliferation, migration, invasion and epithelial‐mesenchymal transition in triple‐negative breast cancer. J Cell Mol Med. 2016; 20: 1640–50. 27027510 10.1111/jcmm.12856 PMC4988287

[17] Lam AP , Herazo‐Maya JD , Sennello JA , *et al* Wnt coreceptor Lrp5 is a driver of idiopathic pulmonary fibrosis. Am J Respir Crit Care Med. 2014; 190: 185–95. 24921217 10.1164/rccm.201401-0079OC PMC4226053

[18] Zhou B , Liu Y , Kahn M , *et al* Interactions between beta‐catenin and transforming growth factor‐beta signaling pathways mediate epithelial‐mesenchymal transition and are dependent on the transcriptional co‐activator cAMP‐response element‐binding protein (CREB)‐binding protein (CBP). J Biol Chem. 2012; 287: 7026–38. 22241478 10.1074/jbc.M111.276311 PMC3293544

[19] Scheraga RG , Thannickal VJ . Wnt/beta‐catenin and transforming growth factor‐beta signaling in pulmonary fibrosis. A case for antagonistic pleiotropy? Am J Respir Crit Care Med. 2014; 190: 129–31. 25025351 10.1164/rccm.201406-1037ED

[20] Wei J , Fang F , Lam AP , *et al* Wnt/beta‐catenin signaling is hyperactivated in systemic sclerosis and induces Smad‐dependent fibrotic responses in mesenchymal cells. Arthritis Rheum. 2012; 64: 2734–45. 22328118 10.1002/art.34424 PMC3553791

[21] Xu L , Li LC , Zhao P , *et al* Total polysaccharide of Yupingfeng protects against bleomycin‐induced pulmonary fibrosis *via* inhibiting transforming growth factor‐beta1‐mediated type I collagen abnormal deposition in rats. J Pharm Pharmacol. 2014; 66: 1786–95. 25209833 10.1111/jphp.12308

[22] Wilson MS , Wynn TA . Pulmonary fibrosis: pathogenesis, etiology and regulation. Mucosal Immunol. 2009; 2: 103–21. 19129758 10.1038/mi.2008.85 PMC2675823

[23] George SJ . Regulation of myofibroblast differentiation by convergence of the Wnt and TGF‐beta1/Smad signaling pathways. J Mol Cell Cardiol. 2009; 46: 610–1. 19233190 10.1016/j.yjmcc.2009.02.008

[24] Henderson WR Jr , Chi EY , Ye X , *et al* Inhibition of Wnt/beta‐catenin/CREB binding protein (CBP) signaling reverses pulmonary fibrosis. Proc Natl Acad Sci USA. 2010; 107: 14309–14. 20660310 10.1073/pnas.1001520107 PMC2922550

[25] Konigshoff M , Kramer M , Balsara N , *et al* WNT1‐inducible signaling protein‐1 mediates pulmonary fibrosis in mice and is upregulated in humans with idiopathic pulmonary fibrosis. J Clin Investig. 2009; 119: 772–87. 19287097 10.1172/JCI33950 PMC2662540

[26] Akhmetshina A , Palumbo K , Dees C , *et al* Activation of canonical Wnt signalling is required for TGF‐beta‐mediated fibrosis. Nat Commun. 2012; 3: 1–12. 10.1038/ncomms1734 PMC3316881 22415826

[27] Tanjore H , Degryse AL , Crossno PF , *et al* beta‐catenin in the alveolar epithelium protects from lung fibrosis after intratracheal bleomycin. Am J Respir Crit Care Med. 2013; 187: 630–9. 23306543 10.1164/rccm.201205-0972OC PMC3733436

[28] He W , Dai C , Li Y , *et al* Wnt/beta‐catenin signaling promotes renal interstitial fibrosis. J Am Soc Nephrol. 2009; 20: 765–76. 19297557 10.1681/ASN.2008060566 PMC2663839

[29] He W , Kang YS , Dai C , *et al* Blockade of Wnt/beta‐catenin signaling by paricalcitol ameliorates proteinuria and kidney injury. J Am Soc Nephrol. 2011; 22: 90–103. 21030600 10.1681/ASN.2009121236 PMC3014038

[30] Lam AP , Gottardi CJ . beta‐catenin signaling: a novel mediator of fibrosis and potential therapeutic target. Curr Opin Rheumatol. 2011; 23: 562–7. 21885974 10.1097/BOR.0b013e32834b3309 PMC3280691

